# Supplementary material for: Patterns of Intron Gain and Loss in Fungi
Source: PLoS Biol. 2004 Nov 30;2(12):e422. doi: 10.1371/journal.pbio.0020422 (PMC532390; doi:10.1371/journal.pbio.0020422)
Supplement: Table S1 — Also available at http://genes.mit.edu/NielsenEtAl/. (4.3 MB ZIP). [file pbio.0020422.st001.zip › NielsenEtAl/html/881.html]

AN3917.1.NCU09115.1.MG09920.1.FG01284.1


```
 CLUSTAL W (1.82) Multiple Sequence Alignments - Introns Inserted


Sequence 1: NCU09115.1	485 aa
Sequence 2: MG09920.1	535 aa
Sequence 3: FG01284.1	533 aa
Sequence 4: AN3917.1	557 aa
Alignment Length: 575 aa
Number Identitical Residues: 178 aa
Alignment Score (without introns) 10696


MG09920.1 	MGLVEVVLEHVSVKAAVLWIVGSWVLYLVVKNVLEERKINKLGARAARMPSRLPLS1---
NCU09115.1	-----------------------------------------MGLRPPKIRDSGLFG1---
FG01284.1 	MGVVEALLEQVTLKTTLVILFVSYCIYSIICRIDEHRRIRRLGHYGPHMKTYAPWG1---
AN3917.1  	--MIEELVSKTTPGQAVVYLLGAFFLACLARKIQVQWQLSRLGSRAPKIPFRLPYG~LLI
          	    .   .  :   :      :                  :*   .::      .    

MG09920.1 	----------------LDNIIRAVRANIQNRNLQMWR-SHMSHSITYTVEGR-------L
NCU09115.1	----------------IKFIKTNVKMAAEHKNLQFWQ-QMFRSIGGYTGEVR-------L
FG01284.1 	----------------LGIVARFVVSTVKHQNLATWRDDMFGAENSWTVETR-------L
AN3917.1  	PSISRNSHADRRLSSAMDFIYKSMQANKSDQDLEYFTDVMRNAKGAPLAEDKKTVELDAG
          	.: : .: :.   ::::  :   :    ..::*  : .           * :.: . .: 

MG09920.1 	LFQRIILTADPENIKAILATQFTDYGKGEPFHQEWKPFLGDSIFATDGEKWHTSRQMLRP
NCU09115.1	VGHRIIFTSEPENMKAILATQFEDYGKGEGFHQEWKDFLGDSIFTTDGDLWHASRQLIRP
FG01284.1 	LGVRTIFTADPANLKAILATQFVDYGKGKPFHAEWKDFLGDSIFTTDGAPWHASRQLIRP
AN3917.1  	ISSRIIFTRDPENIKAILTGQFSDYGKGESFHRDWREFLGDSIFVTDGEQWSASRHLIRP
          	:  * *:* :* *:****: ** *****: ** :*: *******.***  * :**:::**

MG09920.1 	QFVKDRVSDLECFESHIKTLFKAMANGGALEGEHQHVELSAINGKRFEITDLFFRFSLDV
NCU09115.1	QFIKNRVSDLQCFENHMQMLFRTIANGGALNGEDQMVDMEAGNGKPVDISDLFFRYTLDA
FG01284.1 	QFTRDRVSDLHCFEAHMQTLFKAIANRGPLHGEDQPVPTEGLDGKVLDISDLFFRYTLDV
AN3917.1  	MFVRDRLVDTEIFEKHVQHLIPLLAGNN--------------ESKIVDVTSLFFRYTLDA
          	 * ::*: * . ** *:: *:  :*. .              :.* .:::.****::**.

MG09920.1 	ATDFLLGYDVKSLS2TPHAEFAEAFNEVQHVQSTITRSG~PAQALVPKGSYYRGLKVMDN
NCU09115.1	ATDFLLGKDIKSLS2TPVQPFADAFQEVQRVQIVIARAG~PLNRFVPKKTFWEGLKVIDE
FG01284.1 	ATEFLLGWDVKSLT2TPRQEFAEAFNDVQRIQNIVARTG~KLRHIIPRYKFWKGLKTVNH
AN3917.1  	ATNYLLGQGTDSLQ~NPETEFAEAFRYVQRRQAQYFRLG2VFKFVLSRTEFRRQLKVMDD
          	**::*** . .**  .*   **:**. **: *    * *   . .:.:  : . **.::.

MG09920.1 	FINPFIDRALRLSPDELASKTKSDHGYTFLHELASFTRDRKVLRDQIVAVLLAGRDTTAA
NCU09115.1	TINFYIDRALRLDEEELASKSKGDEGYTFLHALAGFTKNRQVLHDQLMAVLLAGRDTTAC
FG01284.1 	FINFYIERALRLSPEELNSKTKDDHSYTFLHALAGFTRDRKVLRDQIIAVLLAGRDTTAA
AN3917.1  	FIKPYIDRVLAMSPAELDQKLSKSD--TFLHALARFTRDRRTLRDQLVAILLAGRDTTAA
          	 *: :*:*.* :.  ** .* . ..  **** ** **::*:.*:**::*:*********.

MG09920.1 	TLSWTIYELGRHPECVKRLRQEIAEVVGF---GRTPTYADLKSMKYLQNVMHETLRL~YP
NCU09115.1	TLSWAIYELARHPEAVAKLRAEILSVVGP---DRAPTYDDLKSMKYLQNVMNETLRL2--
FG01284.1 	TLSWTLYELGRYPNAVKRLRSEILSTLGS---ERTPTYDDLKSMSYLKAVLNETLRL~YP
AN3917.1  	TLSFCLFELARNPEVTAKLREEIASRLGLGASAQKPSYTDLKEMKYLNAVLNESMRL~YP
          	***: ::**.* *: . :** ** . :* .:: : *:* ***.*.**: *::*::**  .

MG09920.1 	AVPFNVRLALHDTTLPRGGGPDGTLPVPVLKDTPIGYSTLLMQRRRDLY-PPVSENFADP
NCU09115.1	-----VRMALKDTTLPRGGGPDGSQPIVILKDTPVGYSPLAMQRRPDLY-PPVSEKFPDV
FG01284.1 	AVPFNVRLALKDTTLPRGGGPDGSEPLPVLKDSPVGYSTLVMQRRSDLY-PPISDTFADP
AN3917.1  	VVPFNVRFSLKDTTLPRGGGPDGLAPVAVRAGTRVVYSTMIMQRSPENYDPPSAPNYFDP
          	  . .**::*:************  *: :  .: : **.: ***  : *.** : .: * 

MG09920.1 	DVFSPERWFH-WQPRPWQYVPFNGGPRICIGQQFALTEMGYVLVRMFQQYERVVSYMDEI
NCU09115.1	EMFSPDRWFH-WQPKPWQYIPFNGGPRICIGQQFALTEMGYVLTRLFQRYDRVVSYMDEI
FG01284.1 	QIFSPERWAH-WHPKPHDYIPFNAGPRICIGQQFALTEMSYVLCRLFQKYERVESRMKDI
AN3917.1  	AQWNPERWLSGWQPKPWQFIPFNGGPRICIGQQFAMIEMGYTVVRILQAFERIQAVPEAG
          	  :.*:**  .*:*:* :::***.***********: **.*.: *::* ::*: :  .  

MG09920.1 	D--GGNPTIKADIVMQPGDGVIVAFWEG----DNEKI--
NCU09115.1	D--GGKPRMKTDIVLMPGDGVKVAFFEA----KRE----
FG01284.1 	D--GGEPLLKADIVLSPGQGVNVAFWEA----KK-----
AN3917.1  	KSVVEDPRLRFEVTLTPASELNCVFVRDGEGREDEKISR
          	.:   .* :: ::.: *.. :  .* . ... . .. :
```
